# HyDEn: A Hybrid Steganocryptographic Approach for Data Encryption Using Randomized Error-Correcting DNA Codes

**DOI:** 10.1155/2013/634832

**Published:** 2013-07-28

**Authors:** Dan Tulpan, Chaouki Regoui, Guillaume Durand, Luc Belliveau, Serge Léger

**Affiliations:** ^1^National Research Council Canada, 100 des Aboiteaux Street, Moncton, NB, Canada E1A 7R1; ^2^Department of Biology, Université de Moncton, Moncton, NB, Canada E1A 3E9; ^3^Department of Computer Science, Université de Moncton, Moncton, NB, Canada E1A 3E9

## Abstract

This paper presents a novel hybrid DNA encryption (HyDEn) approach that uses randomized assignments of unique error-correcting DNA Hamming code words for single characters in the extended ASCII set. *HyDEn* relies on custom-built quaternary codes and a private key used in the randomized assignment of code words and the cyclic permutations applied on the encoded message. Along with its ability to detect and correct errors, *HyDEn* equals or outperforms existing cryptographic methods and represents a promising *in silico* DNA steganographic approach.

## 1. Introduction

The deluge of counterfeited goods flooding the world markets today generates a high demand for novel cryptographic and steganographic approaches that will better protect information and branded products and ensure their authenticity. Positioned at the confluence of mathematics, biology, informatics, chemistry, and physics, cryptography and steganography represent the ultimate means for information protection.

### 1.1. Cryptography

Cryptography is generally defined as the practice and study of techniques for secure communication performed over unsecured channels. There are two major operations involved in secure communication, namely, the encryption and decryption of a message. The purpose of encryption is to modify the information, such that only an authorized party is capable of decoding it. Both, encryption and decryption, require a key, which is needed by the authorized parties, and it is assumed to be kept secret. To date, only one encryption approach was mathematically proven to be secure and virtually unbreakable: the one-time pad [1]. Nevertheless, its practicality is hampered by the necessity of a random key, which must be at least as long as the message itself. For all other cryptographic approaches, there is a theoretical possibility of breaking them, although the time required to do so might be very long, thus making the approaches fairly secure. Examples of such cryptographic approaches include the data encryption standard (DES) [2], the advanced encryption standard (AES) [3], the Rivest-Shamir-Adleman (RSA) method [4], and the Pretty Good Privacy (PGP) [5] method.

### 1.2. Steganography

Steganography is the science of concealing information within different types of media, such that only the sender and the receiver are aware of its exact location. Unlike cryptography, where only the message is protected, steganography protects both the message and the communicating parties. With origins deeply rooted in ancient Greece, where messages were recorded as texts or tattoos and then hidden on wax tablets and skins, steganography was used relentlessly over the centuries under various ingenious forms such as invisible inks [6], postal stamps [7], knitted clothes [8], microdots [9], modified images [10], executable files [11], and DNA sequences embedded in various materials [12, 13].

### 1.3. Error-Correcting DNA Codes

Error-correcting codes consist of sets of symbols defined over a finite alphabet, such that if any code word is altered in *t* positions we can detect and correct the error based on knowledge of the remaining code words.

For example, assume a given binary code *W* consisting of two code words *w*_1_ = 000 and *w*_2_ = 111 each of length 3. A 1-bit error occurring in any of the two code words (e.g., *w*_2_) will produce a modified code word; let us say *w*_2_′ = 101. By comparing the modified code word *w*_2_′ with both code words from *W*, we notice that it differs in only one bit from *w*_2_ (middle bit), while it differs in two bits compared with *w*_1_ (flanking bits). Thus, we can quickly identify the exact location of the error and correct it based on *w*_2_′s closest proximity to code word *w*_2_.

#### 1.3.1. Hamming Codes

 One instance of simple and efficient error-correcting codes are Hamming codes [14], where each pair of code words differs in at least *d* bits. We denote by *A*_4_(*n*, *d*) the size of a quaternary code where all pairs of code words of length *n* differ in at least *d* positions. The number of bits/positions in which two code words differ is also known as the Hamming distance. For certain combinations of *n* and *d*, the exact size of quaternary codes are unknown and thus lower and upper bounds were derived to provide approximations. The text by MacWilliams and Sloane [15] provides a succinct introduction to the topic.

While Hamming codes were originally designed using a {0,1} alphabet with the purpose of sending binary information over noisy channels, the increased need for storing and retrieving information with synthetic DNA strands used as chemical bar codes, or as biological tags for DNA computing applications, facilitated the advent of Hamming codes defined over quaternary alphabets, such as the DNA alphabet {A, C, T, G}.

#### 1.3.2. DNA Codes

A single-stranded DNA molecule is a long, unbranched polymer composed of only four types of subunits linked together by chemical bonds and attached to a sugar-phosphate chain like four kinds of beads strung on a necklace. These subunits are the deoxyribonucleotides containing the bases: adenine (A), cytosine (C), guanine (G), and thymine (T).

Conceptually equivalent to a digital signal, DNA sequences are naturally and synthetically used for information encoding in living organisms and biotechnological and steganographic applications. Given the data encoding capacity of DNA and the fact that traditional data encoding techniques using binary sequences are fortified against communication errors, quaternary codes using the DNA alphabet {A, C, T, G} were proposed and continuously developed over the past decades.

The design of error-correcting DNA codes of fixed length *n* that satisfy various combinations of constraints such as having a minimum pairwise Hamming distance (*d*_min⁡_) is a hard computational problem, whose complexity is still unknown today. Over the past two decades, a large number of publications have proposed intricate code design techniques [16–18] based on their state-of-the-art algorithms such as stochastic local search, genetic algorithms, and pure mathematical constructions. Most of these approaches lead also to the continuous improvement of upper and lower bounds for DNA codes [19–21].

Assuming that a DNA code *C* with *k* code words of length *n* is given and that each pair of distinct code words *w*_*i*_ and *w*_*j*_ obeys the condition that, for all pairs (*w*_*i*_, *w*_*j*_) with *i*, *j* ∈ *N*, *i* ≠ *j*,

(1) $$\begin{array}{c} \text{Hamming}\;\text{Distance}\left(w_{i},w_{j}\right)\geq d, \end{array}$$

then *C* can detect ⌊*d*/2⌋ errors and can correct ⌊(*d* − 1)/2⌋ errors.

### 1.4. Related Work

 Over the past decade, complex algorithms have been devised to encode information using DNA sequences. Examples of such algorithms include the DNA triplet-based approach described by Clelland et al. [9], which extends the principle of using microdots to hide information developed during the Second World War. An extension of Clelland et al.'s work was presented by Leier et al. [22], and it consisted of encoding zeros and ones using short DNA sequences with sticky ends, which can bind together forming longer sequences. The encrypted messages include a mixture of coding and noncoding DNA sequences, and the decryption can be performed only by someone who has access to the correct primer sequences. A primer is a short DNA sequence that serves as a starting point for DNA synthesis. A similar approach based on DNA tiling was proposed by Hirabayashi et al. [23] who designed true random one-time pads using a DNA cryptosystem. The true randomness is conferred by molecular computations using hybridization of DNA sequences encoding 4 types of cipher tiles.

Gehani et al. [24] extended the one-time pad approach to perform operations on DNA sequence pairs, representing plain and cipher texts. Originally, the one-time pad approach was designed to perform XOR operations on binary codes. The message encoded with DNA pairs can be retrieved and decoded using specific DNA polymerases. Arita and Ohashi [25] developed a steganographic algorithm based on the redundant codon table (see Table 1). A codon consists of 3 consecutive nucleotides, and while it is possible to have 64 (4^3^) different codons, only 20 of them encode distinct amino acids, with the rest being redundant. Their algorithm encoded each letter in the English alphabet using binary codes of length 5, with each bit being encoded by a codon. They added an additional parity bit to each letter encoding to keep the number of bits in each bit-pattern odd and thus used for error-detection purposes. The decoding could be achieved only by someone who knows the original codon sequence.

Following a different approach, Wong et al. [27] developed a DNA steganography method that encodes information in living organisms. The information is encoded with the aid of unique DNA sequences that do not exist in the particular genomes where they will be embedded, thus assuring the success of the identification stage. For this approach to succeed, the embedded foreign DNA must be replicated by the host organism together with their genomic DNA. The extraction of the information is achieved using a standard laboratory technique called the polymerase chain reaction (PCR) [28].

The DNA-Crypt approach proposed by Heider and Barnekow [29] combines and extends the steganographic and cryptographic methodologies proposed by Wong et al. [27] and Arita and Ohashi [25]. DNA-Crypt encodes information using a substitution cipher and two types of error-correcting codes, namely, Hamming [14] and WDH [30]. DNA-Crypt incorporates a fuzzy controller and powerful cryptographic algorithms such as one-time pad, AES, Blowfish [31], and RSA. Shiu et al. [32] introduced 3 data hiding methods based on properties of DNA sequences, namely, the insertion method, the complementary pair method, and the substitution method. All three methods provide distinct means to incorporate secret messages within existing DNA sequences pulled from public databases. The known DNA sequence acts as a private key, and it can be identified only by the sender and the receiver.

A hybrid approach built on the substitution method described in Shiu et al. [32] that combines cryptography and DNA steganography was proposed by Torkaman et al. [33]. Their approach uses reference DNA sequences from the European Bioinformatics Institute (EBI) Database, which contains roughly 163 million entries. The encoding of information is achieved using 6 association rules.

Here, we present the hybrid DNA encryption (HyDEn) approach, which combines the advantages conferred by cryptography and steganography into a unique symmetric cryptosystem. The system uses a unique private numeric key to scramble the assignment of DNA code words from a predesigned set to the extended ASCII characters and then apply a cyclic permutation on the encrypted message. The combination of key uniqueness, the randomization of code word assignments, the undisclosed code word length, and the final cyclic permutation of the encrypted message confer additional strength to the proposed approach. The information encrypted with HyDEn can be safely communicated between senders and receivers via dedicated and inconspicuous publicly accessible channels, such as bioinformatics discussion groups and DNA sequence databases.

## 2. HyDEn: The Hybrid DNA Encryption Approach

Deeply rooted in the ways nature encodes information using nucleic acids, DNA stegano-cryptography uses short DNA sequences to encrypt and hide messages, thus protecting their content. The hybrid DNA encryption (HyDEn) approach presented here includes a novel *in silico* cryptosystem that uses DNA error-correcting Hamming codes and disguises encrypted messages as long DNA sequences conveniently placed on host bioinformatics resources.

Following next is a stepwise description of the HyDEn cryptosystem.

*Input*. The message is defined over an alphabet *Ω*, private key *pk*. 

*Encryption Algorithm*

**Step**

Select an error-correcting DNA code with |*Ω* | *n*-ary code words obtained with one of the state-of-the-art code design techniques described in Aboluion et al. [16], Gaborit and King [19], Tulpan and Hoos [26], and Tulpan et al. [18]. Here, *n* represents the number of characters in a DNA code word. An example of a DNA code with *n* = 8 and *d* = 3 is given in Table 2.

**Step**

Using the key *pk* provided as input, perform a random shuffling of the *n*-ary DNA code words that will be associated to each character from *Ω*.

**Step**

Encrypt the message using the random assignment of DNA code words obtained in Step 2.

**Step**

Perform a circular rotation (mod⁡|*Ω*|) to the right of the characters in the message with exactly *pk* positions.

*Output*. The encrypted message *m*.

Step 1 provides the means of encoding a message using a code defined over a quaternary alphabet. The code will be able to identify and correct errors that can occur during the message transmission stage. Step 2 will generate a unique code word assignment based on the key *pk*. If all *pk* keys are unique, then the assignment will be equivalent to a one-time pad system. In the eventuality that code word length (*n*) is found, Step 4 is used to lower the chances of a successful frequency analysis based on well-established tests such as the Friedman test [34] and the Kasiski test [35].

The message decryption step will use the same unique key to perform the reverse circular permutation on the encrypted message and find the correct code words assignment, which will reveal the original message.

**Figure 1:**
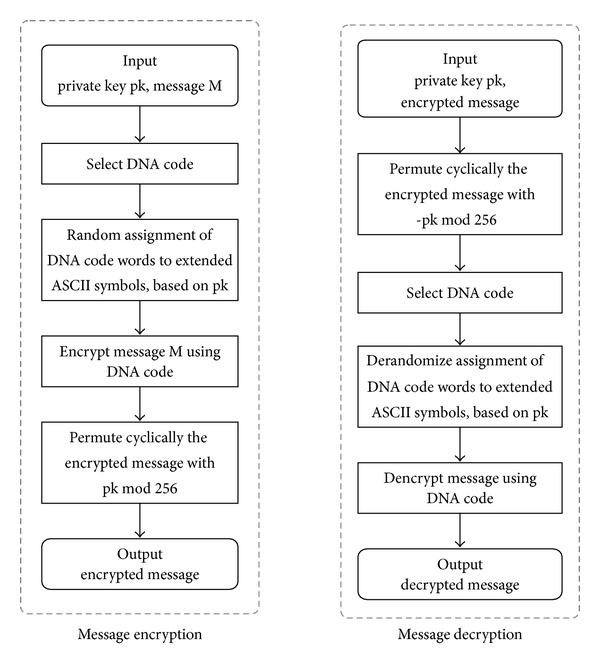
Flowcharts for message encryption and decryption with HyDEn.

The flowcharts for message encryption and decryption with HyDEn are summarized in Figure 1.

## 3. Example of Message Encryption and Decryption Using HyDEn

To better understand how the HyDEn approach works, let us assume that Alice would like to transmit the message “ATTACK AT DAWN” to Bob. They have established before hand to use the secret key “5”. The message uses only 8 distinct ASCII characters, namely, “space,” “A,” “C”, “D,” “K,” “N,” “T” and “W.” Based on the unique key used by Alice and Bob, and applying Steps 1 and 2 of our approach, a unique assignment of DNA code words of length 8 is associated to each of the 8 characters, as shown in Table 3.

 Using this assignment, the encrypted message resulting after Step 3 is the following:

**ACTACACT**GTTGTATT**GTTGTATT**ACTACACT

**ATGGAGTT**CTGGTAGT**AAAAAAGA**ACTACACT 

**GTTGTATT**AAAAAAGA**CCCTTCGA**ACTACACT 

**TCGTGTTA**GGAAAGGT

To better visualize the encryption process, every second code word was bold faced. The encrypted message is then permuted cyclically five positions to the right, thus obtaining the following sequence of DNA bases: 

AAGGT**ACTACACT**GTTGTATT**GTTGTATT**

ACTACACT**ATGGAGTT**CTGGTAGT**AAAAAAGA**

ACTACACT**GTTGTATT**AAAAAAGACCCTTCGA 

ACTACACT**TCGTGTTA**GGA

Ideally, the key (mod 256) must be different from a multiple of the code word length (*n*); otherwise, the permutation will shift the encrypted message exactly *n* letters to the right (or to the left) and will not have the desired effect.

## 4. Comparison Parameters

To facilitate the comparison between our approach and related encryption methodologies, we use a combination of performance parameters including the ones introduced by Shiu et al. [32], namely, capacity, payload, *bpn*, and the cracking probability or the probability of a successful brute-force attack *P*_*bf*_.

The capacity (*C*) is defined as the total length of a reference sequence that encodes or includes the encrypted message. The payload (*P*) is the remaining length of the new sequence after subtracting the reference DNA sequence. The *bpn* represents the number of hidden bits per character. The previous parameters utilize the following notations: *n* is the length of a DNA sequence, *m* is the message that will be encrypted, and |*m*| is its length.

## 5. Results and Discussion

We analyze the robustness of HyDEn by estimating the probability of success for a brute-force attack, and we provide a comparative assessment between our cryptosystem and other cryptographic techniques with performance characteristics described in the literature. The comparison relies on a set of parameters introduced in Section 4. We further investigate HyDEn's strengths and weaknesses, and we provide insights into potential improvements that will augment its performance.

### 5.1. Robustness

Calculations of the strength of encryption against brute-force attacks are typically the worst case scenarios thus, the probability of success for a brute-force attack against the proposed cryptosystem (HyDEn) is captured

(2) $$\begin{array}{c} P_{bf}=\frac{1}{n}\cdot \frac{1}{\left|\Omega \right|!}\cdot \frac{1}{\left|\Omega \right|}, \end{array}$$

where *n* is the length of a DNA code word and |*Ω*| is the number of characters in alphabet *Ω*.

Assuming that *Ω* is the extended ASCII character set, then |*Ω*| = 256 and (2) becomes

(3) $$\begin{array}{c} P_{bf}=\frac{1}{n}\cdot \frac{1}{256!}\cdot \frac{1}{256}. \end{array}$$

Using the Stirling approximation [36] for factorials, ln⁡(*k*!) ≈ *k* · ln⁡(*k*) − *k*, for all *k* ∈ ℝ, and DNA code word length *n* = 8, we obtain

(4) $$\begin{array}{c} P_{bf}\approx \frac{1}{2^{11}\cdot e^{1163.6}}. \end{array}$$

The first term in (2) comes from the fact that *n* is unknown to the attacker; thus, a successful attacker must first guess the length of the used code words, which would be 8 in the sample *A*_4_(8,4) DNA code from Table 2. The second term of the equation describes the probability of finding the correct code assignment for the extended ASCII character set. We also assume that the attacker already knows what character set is encoded by the DNA code. The last term of the equation is given by the probability of finding the correct cyclic permutation applied to the encrypted message. Without knowing the correct permutation, the attempt of identifying the correct code word assignment is prone to failure.

### 5.2. Comparison with Other DNA Cryptographic Strategies

Using the parameter estimations described in Section 4, we compare HyDEn with other encryption approaches described in Shiu et al. [32].

Table 4 presents comparative results between HyDEn and other cryptographic methods. The methods are compared based on their capacity (*C*), payload (*P*), the number of hidden bits per character (*bpn*), and the probability of success for a brute-force attack (*P*_*bf*_).

Based on the probability of success for a brute-force attack (*P*_*bf*_), HyDEn and the insertion method are the most secure, while the substitution method seems to be the least secure. Nevertheless, the best capacity (*C*), payload (*P*), and *bpn* correspond to *HyDEn* and the Substitution method, while the insertion method ranks second and the complementary pair third.

The result expressed in (4) can be also directly compared with the result reported by Torkaman et al. [33] on page 233 in their paper. Their result states that the probability of recovering via a brute-force technique an original message hidden within a sequence database with other 163 million sequences is equal to (1/(1.63 × 10^8^)) × (1/6). Using simple numerical inequality manipulations, we show that our technique confers higher protection against brute-force attacks compared with the method proposed by Torkaman et al.:

(5) 1211×e1163.6 <1211×21163.6<1211×21163=121174 ≪1232=124×228=12×23×228<12×6×228 <12×6×108<11.63×108×16.

Thus, *P*_*bf*_  (HyDEn) ≪ *P*_*bf*_ (substitution: Torkaman et al. [33]).

### 5.3. HyDEn's Strengths, Weaknesses, and Potential Extensions

 Compared with the existing DNA-based cryptographic and steganographic methods, HyDEn has one of the lowest probabilities of success for brute-force attacks. HyDEn includes mechanisms such as cyclic permutations and randomized assignments of code words to protect against various types of frequency analysis such as the Kasiski and Friedman tests along with error detection and correction capabilities conferred by DNA Hamming codes. One of the drawbacks of using many-to-one character encoding schemes is the increase in size of the encrypted message, which could become a burden for the communication media and which also poses also a challenge for hiding strategies of large messages. The steganographic approach including message distribution and the selection of inconspicuous dissemination venues must be carefully analyzed. For example, large encrypted messages encoded as long *in silico* DNA sequences can be better hidden in databases for DNA coding sequences, DNA contigs or mRNA sequences, while relatively short messages would be better hidden as DNA and RNA primer sequences or as microarray probes.

One potential weakness of the current approach could stem from peculiarities of the language in which the original message was written, assuming that the attacker has already guessed it. For example, if English is the language, then an analysis based on occurrences of double letters such as double Ls in a fairly limited number of words could be used to find partial (code word, character) associations. A potential extension inspired from the Belasso Ciphers [37], which were later wrongfully attributed to Vigenère [38], that will add confusion and increased security to HyDEn is to encode each character with multiple code words selected uniformly at random, without breaking the error detection and correction capabilities of the DNA code.  Table 5 presents an *A*_4_(8,3) code with 1024 DNA sequences of length 8 and minimum pairwise Hamming distance 3, which could be used as a replacement of the code from Table 2. Each extended ASCII character could be encoded using one out of 4 different code words, each selected with equal probability. Lower (2048) and upper (2340) bounds published by Bogdanova et al. [39] and hosted on Dr. Andries Brower's website [40] suggest that even larger *A*_4_(8,3) DNA codes can be generated.

## 6. Conclusion

Here, we have presented a novel stegano-cryptographic approach called HyDEn (hybrid DNA encryption), which uses custom-built error-correcting DNA Hamming codes, a randomized code assignment procedure and cyclic permutations based on a private key. HyDEn represents a symmetric cipher that is capable of encrypting and disguising information as long DNA sequences in public bioinformatics discussion groups and DNA sequence databases. Our cryptosystem has significant error tolerance and adds another dimension to the information security field. We are currently working on experimentally evaluating and further improving HyDEn's capabilities following the ideas described in Section 5.3.

## Figures and Tables

**Table 1:** The redundant DNA codon table.

Amino acid	DNA codons					
Alanine	GCT	GCC	GCA	GCG		
Arginine	CGT	CGC	CGA	CGG	AGA	AGG
Asparagine	AAT	AAC				
Aspartic acid	GAT	GAC				
Cysteine	TGT	TGC				
Glutamic acid	GAA	GAG				
Glutamine	CAA	CAG				
Glycine	GGT	GGC	GGA	GGG		
Histidine	CAT	CAC				
Isoleucine	ATT	ATC	ATA			
Leucine	CTT	CTC	CTA	CTG	TTA	TTG
Lysine	AAA	AAG				
Methionine	ATG					
Phenylalanine	TTT	TTC				
Proline	CCT	CCC	CCA	CCG		
Serine	TCT	TCC	TCA	TCG	AGC	AGT
Threonine	ACT	ACC	ACA	ACG		
Tryptophan	TGG					
Tyrosine	TAT	TAC				
Valine	GTT	GTC	GTA	GTG		
Start (CI)	ATG					
Stop (CT)	TAA	TAG	TGA			

**Table 2:** A sample DNA *A*_4_(8,3) Hamming code consisting of 256 code words. Each code word can be associated with an extended ASCII character and used for encoding text messages. The code was obtained with the DNA word design algorithm described in Tulpan and Hoos [26].

A set with 256 code words							
AAAAAAGA	ACTACACT	ATGGAGTT	CCCTTCGA	CTGGTAGT	GGAAAGGT	GTTGTATT	TCGTGTTA
AAAAGAAG	ACTACCTA	ATGGGAAG	CCGATTTC	CTGGTTCG	GGATGACA	TAACATAC	TCTCCGAG
AAAATGTT	ACTCTCAG	ATGTAAGT	CCGCGCAT	CTTCGGTG	GGCCAAGT	TAACCATA	TCTCCTTA
AAACCTGC	ACTGGAGT	ATTCATAC	CCGGCGCG	CTTGACAT	GGCCGACG	TAACGAGG	TCTGCGCA
AAACTCAC	ACTTCCGC	ATTCTGCG	CCGTAGCC	CTTGCATG	GGCCTGGA	TAAGAGCA	TCTGGCTC
AAAGATCG	ACTTGCAT	ATTTAATC	CCGTTCAG	CTTTCCAC	GGCGTGCC	TAAGTTGA	TCTGTTAC
AAATGTGG	ACTTTGGG	ATTTCAGA	CCTACCGG	GAATCATC	GGCTGCAT	TAATAGGC	TGAAAATA
AAATTGAG	AGACCCTA	CAAATACG	CCTTCTGT	GACAGCGT	GGGCATAC	TAATGGAA	TGACTCAT
AACAGCTG	AGACTTAA	CAAATCTA	CCTTGTCG	GACCAGCT	GGGCTTGG	TAATTACT	TGAGCATC
AACCTAGC	AGAGCGGT	CAATATGA	CCTTTGAC	GACCGTTA	GGGGCCCA	TACGCAAA	TGAGGGTT
AACGCGTT	AGAGTAAT	CAATTCGC	CGAACGCT	GACGGTAT	GGGGGTTC	TACTTGGG	TGATATAT
AACGGTGA	AGATCTTG	CACCTAAT	CGACCTTT	GAGAATTA	GGTAATGG	TAGACTGA	TGATTCGG
AACTACGT	AGATGGCT	CACTCGAA	CGAGAAAC	GAGAGAGC	GGTACGTA	TAGAGTAC	TGCATAAG
AACTCATA	AGCCAGCA	CAGACAGG	CGAGCGTA	GAGAGTCG	GGTATGCG	TAGGAGTG	TGGGGCGC
AAGAAACT	AGCTCGGG	CAGCAACG	CGAGCTCG	GAGTTGTT	GGTTTAGT	TAGTAACC	TGGTTTTT
AAGATAAC	AGGACTGT	CAGCCGGC	CGAGTCTT	GATACCCC	GGTTTCCC	TAGTCCGG	TGTCAGAT
AAGCACGC	AGGATGAG	CAGGTCGA	CGATGTAC	GATATTGC	GTAACGCG	TATAAATG	TGTGCAAT
AAGGTTGT	AGGCCCAT	CAGTGATC	CGCCACGA	GATCATAT	GTACTACG	TATATGGT	TGTGTTGG
AATAGTCT	AGGTACTT	CATCGAGC	CGCCTCCC	GATCCCAG	GTAGATCA	TATGTGAA	TTAAGCCG
AATCGTTC	AGGTAGGC	CATCTTTG	CGGAAGTA	GATGACTA	GTAGTCGT	TCAAACGC	TTAATTTA
AATGCGGG	AGGTGTCC	CATGCTTA	CGGTAACA	GATTGTTG	GTCATATG	TCAAAGTG	TTAGCTGT
AATGTGCT	AGTCGAAG	CATGGGGA	CGGTGTTG	GATTTACG	GTCCGAAT	TCAAGAAC	TTAGTCCA
AATTGGTT	AGTCGGGA	CCACCGCC	CGTCACAC	GCAGGTCG	GTCCTTAA	TCACAAGA	TTCAAGAC
ACACTAGT	AGTGCCGA	CCAGATGC	CGTTAGCT	GCATTCTT	GTCGCAAG	TCAGTGCC	TTCCGCAC
ACACTTCC	ATATGCCC	CCAGTATC	CTAACTCC	GCATTTCA	GTCTCCAA	TCATCTTC	TTCGAATA
ACAGCTTA	ATCACAAA	CCAGTGGA	CTAGACGG	GCCGAATT	GTGGAGAA	TCCGAGGC	TTGCGTTC
ACATCGAA	ATCACCGG	CCATGACC	CTAGAGCC	GCCGCGGT	GTGGCCAT	TCCGCCGA	TTGGGGTA
ACCGGATC	ATCCCTGA	CCATGCAA	CTATTACA	GCGAATGT	GTGTCGGT	TCCTGAAG	TTGTCTTG
ACCTCAAC	ATCGTAGG	CCCCTACG	CTATTGTT	GCGACATT	GTTATCAC	TCGATGCG	TTTACAGC
ACGCATTT	ATCTCTTC	CCCGGAGA	CTCCCAGT	GCGGGTAA	GTTCACTG	TCGGAACA	TTTCCACG
ACGCTATG	ATCTTCAC	CCCGGGAG	CTCCGGCC	GCTGAGTG	GTTCCAAC	TCGTAGAG	TTTCGTAG
ACGTCGTC	ATGACGTG	CCCTAGTT	CTCGCGGC	GCTGTCCG	GTTGCTCT	TCGTCCAT	TTTGTGTG

**Table 3:** A sample assignment of code words to ASCII characters.

DNA code word	ASCII character
AAAAAAGA	→space
ACTACACT	→A
ATGGAGTT	→C
CCCTTCGA	→D
CTGGTAGT	→K
GGAAAGGT	→N
GTTGTATT	→T
TCGTGTTA	→W

**Table 4:** Comparison between *HyDEn* and other encryption methods. *n* is the length of a DNA sequence, |*m*| is the length of the original message, |Ω| is the size of the DNA code, and *k* is a method-specific parameter that represents the length of the longest complementary pairs in the reference DNA sequence.

Method	*C*	*P*
*Hy* *DE* *n*	*n*	0
Insertion [32]	$n+\frac{\left|m\right|}{n}$	$\frac{n}{2}$
Complementary pair [32]	*n* + |*m* | ·(*k* + 3.5)	|*m* | ·(*k* + 3.5)
Substitution [32, 33]	*n*	0

Method	*bp* *n*	*P*_bf_

*Hy* *DE* *n*	$\frac{\left|m\right|}{n}$	$\frac{1}{n}\cdot \frac{1}{|\Omega |!}\cdot \frac{1}{\left|\Omega \right|}$ (e.g., $\frac{1}{2^{11}\cdot e^{1163.6}}$)
Insertion [32]	$\frac{\left|m\right|}{n+|m|/2}$	$\frac{1}{1.63\cdot 10^{8}}\cdot \frac{1}{n-1}\cdot \frac{1}{2^{\left|m\right|}-1}\cdot \frac{1}{2^{n}-1}\cdot \frac{1}{24}$
Complementary pair [32]	$\frac{\left|m\right|}{n+|m|\cdot (k+3.5)}$	$\frac{1}{1.63\cdot 10^{8}}\cdot \frac{1}{24^{2}}$
Substitution [32, 33]	$\frac{\left|m\right|}{n}$	$\frac{1}{1.63\cdot 10^{8}}\cdot \frac{1}{6}$ or 3^*n*^

**Table 5:** A sample DNA *A*_4_(8,3) Hamming code consisting of 1024 code words. Four distinct code words can be associated with one extended ASCII character and used for encoding text messages. The code was obtained with the DNA word design algorithm described in Tulpan and Hoos [26].

A set with 1024 code words							
AAAAAAAG	AAAAAGGA	AAAACTCC	AAAAGCAC	AAACAATA	AAACAGCT	AAACCGTC	AAACGAGG
AAACGCCA	AAACGTAT	AAAGATGG	AAAGTCTT	AAAGTGGC	AAATATAA	AAATCTTT	AAATGGCG
AAATTGAT	AACACAAA	AACAGACC	AACAGCTA	AACCAGGG	AACCATCA	AACCCTAC	AACCTCGA
AACCTGTT	AACGACCG	AACGCATC	AACGGGGA	AACGTTAA	AACTCGTG	AACTGTGC	AAGAAGAC
AAGACTAG	AAGAGGTG	AAGATACA	AAGATGGT	AAGATTTC	AAGCGTGA	AAGCTCAT	AAGCTGCC
AAGGACGC	AAGGCCTG	AAGGCGCA	AAGGGATA	AAGGTAAG	AAGTAATT	AAGTGCAG	AATAATTA
AATACACG	AATACCTT	AATCAACC	AATCCGGA	AATCGCTC	AATGGAGC	AATGGCAA	AATGGTTG
AATGTGCT	AATTACTG	AATTAGCA	AATTCAAC	AATTGGGT	AATTTAGG	AATTTTCC	ACAAAGTC
ACAACCCG	ACAAGAGT	ACAATTTT	ACACATTG	ACACCAAT	ACACCTCA	ACACTACG	ACACTGTA
ACAGCTGC	ACAGGGCC	ACAGGTAG	ACATAACT	ACATACGA	ACATCATG	ACATTCAC	ACCAACCA
ACCACTGA	ACCAGTTG	ACCATGAC	ACCCAATT	ACCCCCGG	ACCCGAAG	ACCCGGCT	ACCGACAC
ACCGAGTA	ACCGCGAT	ACCGTAGC	ACCTATCG	ACCTGACA	ACCTGCTC	ACCTTTAT	ACGAATGG
ACGACGCC	ACGAGGGA	ACGATCAG	ACGCAACA	ACGCCCAA	ACGCCGTT	ACGCGTAC	ACGCTGGG
ACGGCCGT	ACGGTTCT	ACGTAGAA	ACGTTAGT	ACTAAATG	ACTACGAA	ACTAGCCC	ACTATCTA
ACTATTGC	ACTCATGT	ACTGCACC	ACTGCTTT	ACTGGTCA	ACTGTCAT	ACTTATTC	ACTTCCAG
ACTTCGCT	ACTTGTGG	ACTTTAAA	ACTTTGTG	AGAACCAT	AGAATCTC	AGAATGCG	AGACAAAC
AGACGCGT	AGACGTTA	AGACTCAA	AGAGAGCA	AGAGCACT	AGAGCCGA	AGAGTATA	AGAGTTCC
AGATCGAC	AGATGAGC	AGCAACGT	AGCAATAA	AGCACTTC	AGCAGGCA	AGCATAAT	AGCCAGAT
AGCCCACA	AGCCGTGG	AGCGCCCC	AGCGGGAC	AGCGTCAG	AGCTAAGA	AGCTATTT	AGCTCAAG
AGCTGCAA	AGCTTGGT	AGGAACTG	AGGACATT	AGGAGTGC	AGGATGTA	AGGCAAGG	AGGCATCC
AGGCCTTG	AGGGACAT	AGGGCAAA	AGGGCGGC	AGGGGACC	AGGGGGTT	AGGTAGTC	AGGTCGCG
AGGTCTGT	AGGTGTCA	AGGTTCCC	AGGTTTAG	AGTAACAC	AGTAGGTC	AGTATCCT	AGTCAGTG
AGTCCAGT	AGTCCGCC	AGTGATCG	AGTGCTAC	AGTGGAAT	AGTGGCGG	AGTGTGGA	AGTTCCTA
AGTTGACG	AGTTTATC	ATAAACTT	ATAACATA	ATAATTCA	ATACCCAG	ATACGGTT	ATACTTGC
ATAGAAGT	ATAGCGTG	ATAGGTTC	ATAGTGAA	ATATAGGC	ATATCCTC	ATATCTGG	ATATGCCT
ATCAAGTG	ATCACGGC	ATCAGCCG	ATCAGTAC	ATCATTGT	ATCCAGCC	ATCCATAG	ATCCCCCT
ATCCCTTA	ATCCTAAA	ATCGAACA	ATCGATGC	ATCGCAGG	ATCGTGTC	ATCGTTCG	ATCTAATC
ATCTACAT	ATCTCTCC	ATCTGGAG	ATCTTCGC	ATCTTGCA	ATGAAACT	ATGAGATC	ATGAGCGT
ATGCATTT	ATGCCAAC	ATGCGGAA	ATGCGTCG	ATGGACTA	ATGGAGGG	ATGGGCAC	ATGGTAGA
ATGTACCG	ATGTCGGA	ATGTGAGG	ATTAAAAA	ATTAAGGT	ATTACCCA	ATTAGTCT	ATTATTAG
ATTCCATG	ATTCCCGC	ATTCGACA	ATTGACCT	ATTGAGAC	ATTGCTGA	ATTGGGCG	ATTGTATT
ATTTCTAT	ATTTGCGA	CAAAATCA	CAAACTGG	CAAAGGAA	CAAATCCC	CAACACTC	CAACCCAT
CAACGGCC	CAAGAATT	CAAGCCTA	CAAGCTCT	CAAGGCCG	CAAGGGGT	CAATACAG	CAATATGT
CAATGTTG	CAATTGCA	CACAAAAC	CACAAGTA	CACACCCG	CACATTCT	CACCAACG	CACCGCGG
CACCTTTC	CACGCAGT	CACGCTTG	CACTACGC	CACTAGAT	CACTCTCA	CACTGAGA	CACTTTAG
CAGAAATG	CAGACCAC	CAGACGCT	CAGAGCCA	CAGAGTGT	CAGCACGA	CAGCATAT	CAGCCTCG
CAGCGAAC	CAGCTAGT	CAGGAGTC	CAGGCTGC	CAGGGTAA	CAGTATTA	CAGTCACC	CAGTCCGT
CATAGCAT	CATAGTTC	CATATAAG	CATATGTT	CATCACCT	CATCCTTT	CATCTCAC	CATCTGCG
CATCTTGA	CATGAGGG	CATGATAC	CATGCGAT	CATGTACC	CATGTCTG	CATTCATA	CATTCGGC
CATTGGAG	CATTGTCT	CCAAACTA	CCAAAGGG	CCAACAAC	CCAACGTT	CCACAAAG	CCACCCGA
CCACGTGG	CCACTGAC	CCAGAAGA	CCAGATCG	CCAGGGTG	CCAGTTTC	CCATAATC	CCATCAGT
CCATCGCC	CCATCTAG	CCATGCAT	CCATGGGA	CCATTCTG	CCCAATAT	CCCACATG	CCCAGCCT
CCCAGTGC	CCCATTTA	CCCCACCC	CCCCATGA	CCCCCGAA	CCCCGGTC	CCCCTAGG	CCCGACGT
CCCGCGGC	CCCGGCTA	CCCGTATT	CCCGTCCG	CCCTCTTC	CCCTTGCT	CCGAGTAG	CCGATAAT
CCGATGCG	CCGCAGGC	CCGCCCTC	CCGCGAGA	CCGCGGAT	CCGCTTCA	CCGGAAAC	CCGGCGAG
CCGGCTTA	CCGGTCGA	CCGTACCA	CCGTCAAA	CCGTCTCT	CCGTGATG	CCGTGCGC	CCGTTGTA
CCTAAAGC	CCTACTCA	CCTAGACG	CCTAGGAC	CCTATGGA	CCTCAATA	CCTCCCCG	CCTCCTAC
CCTCGGCA	CCTCTCGT	CCTGAGCT	CCTGCTGG	CCTGGATC	CCTTCCTT	CGAAAAGT	CGAAACCG
CGAACGCA	CGAAGTAC	CGACATCT	CGACCGGG	CGACTAGA	CGACTGTT	CGACTTAG	CGAGAGAT
CGAGATGC	CGAGCATG	CGAGCTAA	CGATAGTG	CGATGAAG	CGATGCTA	CGATTCGT	CGCACGAG
CGCAGAAA	CGCAGCTG	CGCATACC	CGCCAAGC	CGCCACTT	CGCCCGCT	CGCCGTCA	CGCCTTGT
CGCGGACT	CGCGGTTC	CGCGTCGC	CGCTCGTA	CGCTCTGG	CGCTGTAT	CGCTTCCA	CGCTTGAC
CGGACAGA	CGGACGTC	CGGATCAA	CGGCACAC	CGGCGGTG	CGGCTCCG	CGGGAACA	CGGGCCTT
CGGGGTGG	CGGGTATC	CGGGTTAT	CGGTAGGA	CGGTCCAG	CGGTGGCC	CGGTTACT	CGTAAGCC
CGTACCGT	CGTATTCG	CGTCAGAA	CGTCCATC	CGTCGCGA	CGTCTGGC	CGTGATTA	CGTGCGCG
CGTGGCAC	CGTGTAGT	CGTTAAAC	CGTTCTCC	CGTTGGTT	CTAACTTC	CTAAGACT	CTAATGGC
CTACACGT	CTACATAA	CTACCGTA	CTACGAGC	CTACGGAG	CTACTCCA	CTAGCCAC	CTAGGATA
CTATAGCT	CTATGGTC	CTATTAAA	CTATTTTT	CTCAACGG	CTCAATCC	CTCACTAA	CTCATCAT
CTCCCCTG	CTCCGGGT	CTCGACTC	CTCGCCCA	CTCGGTAG	CTCGTAAC	CTCTCATT	CTCTGCAC
CTCTTGTG	CTGAAGAA	CTGAGGTT	CTGATCTG	CTGCAGCG	CTGCGCCC	CTGCGTTA	CTGCTAAG
CTGGATGT	CTGGCAAT	CTGGCCGG	CTGGGGGA	CTGGTCCT	CTGTACTT	CTGTATAC	CTGTTAGC
CTGTTTCG	CTTAATTT	CTTACGTG	CTTAGCGC	CTTATATC	CTTCACAG	CTTCGTAT	CTTCTACT
CTTCTTTG	CTTGAATG	CTTGACGA	CTTGCAGC	CTTGGCTT	CTTGGTCC	CTTGTGTA	CTTTATCA
CTTTCGAA	CTTTGAGT	CTTTGCCG	CTTTTGCC	GAAAACGT	GAAACCTG	GAAACGGC	GAAAGTGA
GAAATATA	GAAATTAT	GAACAGTG	GAACGAAA	GAAGAACG	GAAGCTAG	GAATACCC	GAATCGAA
GAATGGTT	GAATTACT	GAATTCGG	GACAAGCG	GACATCAA	GACATTTG	GACCAAAT	GACCCCGT
GACCGGTA	GACGACGA	GACGATCC	GACGCGAC	GACGGACA	GACGGCAG	GACGTCCT	GACTAATA
GACTCCTC	GACTCTAT	GACTTGGC	GAGAACAG	GAGACCGA	GAGAGAGG	GAGAGTCC	GAGCCTTA
GAGCGGAG	GAGCTCCA	GAGCTTGC	GAGGCCCC	GAGGGGCT	GAGGTATT	GAGGTGGA	GAGTGCTA
GAGTTAAC	GAGTTGCG	GATAAACT	GATAGGCA	GATATCTC	GATCATTC	GATCCACA	GATCGCCG
GATCGGGC	GATCTATG	GATCTGAT	GATGAGTA	GATGGCGT	GATGTAAA	GATTAAAG	GATTCCCT
GATTCTTG	GATTGATC	GCAAACAC	GCAAGATG	GCAATCCT	GCAATGAA	GCACATAT	GCACCAGC
GCACCGCG	GCACGCAG	GCACGGGT	GCACTATT	GCAGCAAA	GCAGGCGC	GCAGGTCT	GCATAGCA
GCATATGG	GCATTGTC	GCCAAAAA	GCCACTCC	GCCAGGAT	GCCATCGC	GCCCAGAC	GCCCGTCG
GCCCTCAT	GCCGCTGT	GCCGGAGG	GCCGGTAC	GCCGTGAG	GCCTACTT	GCCTCACG	GCCTCGGA
GCCTGGCC	GCCTTTCA	GCGAAACG	GCGAGAAC	GCGAGTTA	GCGATAGA	GCGCAATC	GCGCACGT
GCGCCAAG	GCGGGCAT	GCGGGGTC	GCGGTTGG	GCGTATCC	GCGTCCTG	GCGTGACT	GCGTGGGG
GCGTTGAT	GCTACAGT	GCTACGTC	GCTACTAG	GCTAGCGG	GCTCACTG	GCTCCTGA	GCTCGTTT
GCTGAATT	GCTGACAA	GCTGAGGC	GCTGCGCA	GCTTCCGC	GCTTTCCG	GCTTTTAC	GGAACAAG
GGAACTGT	GGAAGGGG	GGAATAGC	GGACACGA	GGACCATA	GGACCGAT	GGACTCTG	GGAGATTT
GGAGCCCG	GGAGGAGT	GGAGGGTA	GGAGTTGA	GGATAAAA	GGATCTTC	GGATGCAC	GGATTGAG
GGCAATCT	GGCACCGG	GGCAGTAG	GGCATGGA	GGCCCCAA	GGCCCTTT	GGCCTAAC	GGCGAATC
GGCGATGG	GGCGCAAT	GGCGGGCG	GGCGTGTT	GGCTATAC	GGCTGATT	GGCTTAGG	GGGAAATA
GGGAAGAT	GGGACCCT	GGGACTAC	GGGCAGCA	GGGCATAG	GGGCGCGG	GGGCGTTC	GGGCTGGT
GGGGCGTG	GGGGCTCA	GGGGTCAC	GGGTAAGT	GGGTGGAA	GGGTTTTA	GGTAATTG	GGTAGACC
GGTAGCTA	GGTATGAC	GGTCAACG	GGTCACAT	GGTCGGCT	GGTCTCCC	GGTCTGTA	GGTCTTGG
GGTGCCTC	GGTGCGGT	GGTGGATG	GGTGGTGC	GGTGTTCT	GGTTACCA	GGTTAGGG	GGTTCTAA
GGTTTAAT	GTAAAATC	GTAATGTG	GTACCCCC	GTACGGCA	GTACTTTA	GTAGACAT	GTAGAGCC
GTAGCGGA	GTAGGTAA	GTAGTACA	GTAGTCTC	GTATCCGT	GTATCTCA	GTATGACG	GTATGTGC
GTCACAAC	GTCACGTT	GTCAGCTC	GTCAGTCA	GTCCAAGA	GTCCCGAG	GTCCGATG	GTCCTCCG
GTCGAGAA	GTCGCATA	GTCGGTTT	GTCGTAGT	GTCTAACT	GTCTATTG	GTCTGAAA	GTCTGCGG
GTGAAGGC	GTGAGGCG	GTGATACC	GTGATTAA	GTGCACAA	GTGCCAGT	GTGCCGTC	GTGCGCTT
GTGCTTCT	GTGGAAAG	GTGGATTC	GTGGGAGC	GTGTAGTA	GTGTCCAC	GTGTCTTT	GTGTGTAG
GTGTTATG	GTGTTCGA	GTTAACCC	GTTAAGAG	GTTACCAT	GTTACTGC	GTTAGATT	GTTATGCT
GTTCAGTT	GTTCCCTA	GTTCCTCG	GTTCGCAC	GTTCTAGC	GTTGCACT	GTTGGCCA	GTTGGGAT
GTTGTCGG	GTTTATGT	GTTTCAGA	GTTTGGTG	GTTTTCTT	TAAAATTG	TAAACAAT	TAAAGACG
TAAAGGTC	TAACCGAG	TAACGTGC	TAACTACC	TAACTGGT	TAAGACCT	TAAGAGAC	TAAGCTTC
TAAGGAGA	TAAGTCAA	TAAGTGTG	TAATACTA	TAATCCAC	TAATCTCG	TAATTAAG	TACAAACA
TACAACTC	TACAGGAG	TACATAGT	TACCATGT	TACCCAGA	TACCTGAA	TACGAAGC	TACGCAAG
TACGGTCT	TACGTCGG	TACGTGCC	TACTCGCT	TACTGATG	TACTGCCA	TACTTCTT	TAGACATC
TAGATCCG	TAGCACTG	TAGCGATT	TAGCTTAG	TAGGAAAT	TAGGAGCG	TAGGTCTC	TAGGTTGT
TAGTAAGA	TAGTCGGG	TAGTGGAT	TAGTGTTC	TAGTTTCA	TATAAGAT	TATAATGC	TATACGTA
TATACTCT	TATATTAA	TATCAAAA	TATCCCCC	TATCCTGG	TATCGGTG	TATCGTCA	TATGACAG
TATGCATT	TATGCCGA	TATTATTT	TATTGCGG	TATTTGTC	TCAAATGA	TCAACCGC	TCAAGAAA
TCAAGGCT	TCAATTCG	TCACAAGT	TCACACAA	TCACGATC	TCACTCGG	TCAGAACC	TCAGAGTT
TCAGCCTG	TCAGTAAT	TCAGTGCA	TCATCCCT	TCATCTTA	TCATGGAG	TCATTTGT	TCCAAGGC
TCCACACT	TCCATCTG	TCCCCCTT	TCCCCTAG	TCCCGCGC	TCCGATTC	TCCGGGAA	TCCGTGGT
TCCTACAG	TCCTCGAC	TCCTGTTT	TCCTTAGA	TCGAAATT	TCGACCCA	TCGAGCTC	TCGATCGT
TCGATTAC	TCGCCATA	TCGCCTGT	TCGCGGCC	TCGGACGG	TCGGATCA	TCGGCACG	TCGGCCAC
TCGGCGGA	TCGGGTGC	TCGTAGTG	TCGTATAT	TCGTGCCG	TCGTTATC	TCTACGCG	TCTAGTAT
TCTATAGG	TCTCAGAG	TCTCATCC	TCTCCGGC	TCTCTAAC	TCTCTGCT	TCTGCTAA	TCTGGACT
TCTGTCGC	TCTGTTTG	TCTTAACA	TCTTACGT	TCTTCAAT	TCTTGCAC	TCTTGGTA	TGAAAGTA
TGAAGTTT	TGAATCGA	TGAATGAT	TGACCACG	TGACGCCC	TGACGGAA	TGACTTCA	TGAGCAAC
TGAGGGGC	TGAGGTCG	TGATACGG	TGATAGCC	TGATCGTT	TGATGACT	TGATTTAC	TGCAAATG
TGCACTAT	TGCAGCAC	TGCAGTGA	TGCCAGCG	TGCCCTGC	TGCCGAAT	TGCCTCTC	TGCGACAA
TGCGCGGG	TGCGCTTA	TGCGGCTT	TGCGTACG	TGCTCACC	TGCTGGTC	TGCTTTCT	TGGACGAA
TGGACTGG	TGGATAAG	TGGCATTA	TGGCCCAT	TGGCGAGC	TGGCGTCT	TGGCTGAC	TGGGAGGT
TGGGGCGA	TGGGGGAG	TGGTACCT	TGGTCCGC	TGGTGATA	TGGTTCTG	TGTAACTT	TGTACACA
TGTAGAGT	TGTAGCCG	TGTCACGC	TGTCCCTG	TGTCGTAC	TGTCTATT	TGTGAAGA	TGTGCCCT
TGTGGGCA	TGTGTCTA	TGTTATAG	TGTTCAGG	TGTTTGCG	TGTTTTGA	TTAACCAA	TTAAGCGG
TTAAGTCC	TTACAACA	TTACAGTC	TTACCATT	TTACCTGA	TTACGTTG	TTACTTAT	TTAGACGC
TTAGATTA	TTAGCGCT	TTAGTAGG	TTATAAAT	TTATCAGC	TTATTCCG	TTATTGTA	TTCACTTG
TTCAGAGC	TTCATATA	TTCATGCG	TTCCAAAC	TTCCCGCA	TTCCGCAA	TTCCTTGG	TTCGAATT
TTCGCCAT	TTCGGCCC	TTCGGGTG	TTCTAGGG	TTCTATAA	TTCTCCTA	TTCTCTGT	TTCTGTCG
TTCTTGAT	TTCTTTTC	TTGAACGA	TTGACGGT	TTGAGGAC	TTGATTTT	TTGCAGAT	TTGCATGC
TTGCCCCG	TTGCGGGG	TTGCTCTA	TTGGCTCC	TTGGGTAT	TTGGTCAG	TTGGTGGC	TTGTAACC
TTGTCAAG	TTGTGTGA	TTTAATCG	TTTAGTTA	TTTATAAT	TTTCAAGG	TTTCCTTC	TTTCGCGT
TTTCTGGA	TTTGCGAG	TTTGGAAA	TTTGGTGG	TTTGTTAC	TTTTACTC	TTTTGGCT	TTTTTCAA
